# Incidence and Radiological Predictors of Concomitant Meniscal and Cruciate Ligament Injuries in Operative Tibial Plateau Fractures: A Prospective Diagnostic Study

**DOI:** 10.1038/s41598-018-31705-x

**Published:** 2018-09-06

**Authors:** Hengrui Chang, Zhanle Zheng, Decheng Shao, Yiyang Yu, Zhiyong Hou, Yingze Zhang

**Affiliations:** 1 Department of Orthopaedic Surgery, the Third Hospital of Hebei Medical University, Shijiazhuang, Hebei, 050051 P.R. China; 2 Key laboratory of biomechanics of Hebei Province, Shijiazhuang, Hebei 050051 P.R. China

## Abstract

The aim of this prospective study was to determine the incidence of meniscal and cruciate ligament injuries in operative tibial plateau fractures detected using knee arthroscopy, and to identify the radiological predictors observed on CT images. From January 2016 to February 2017, a total of 102 closed tibial plateau fractures were enrolled in this prospective protocol. Each patient underwent arthroscopic examination following the tibial plateau internal fixation. Univariate analysis and multivariable logistic regression were used to assess the association between imaging parameters and soft-tissue injuries. The menisci were traumatically injured in 52.9% of subjects (54 of 102) and the cruciate ligaments injured in 22.5% (23 of 102). Significantly higher injury rates for bucket-handle meniscal tears were observed in Schatzker type VI fractures (*P* = 0.04). Greater risk of lateral meniscus injury was observed in patients with >6.3 mm of lateral joint depression. Greater risk of ACL injury when the volumetric lateral joint depression was ≤209.5 mm^2^ and/or with >5.7 mm lateral joint widening. Associated meniscal and ligament injuries were commonly seen among operative tibial plateau fractures. Preoperative CT measurements might help predict a higher risk of meniscus and ACL injury, providing guidance to the surgeon to look for and to be prepared to treat such injuries.

## Introduction

Tibial plateau fractures are complex traumatic injuries that can be quite challenging to reduce and stabilize, and are likely to develop postoperative soft tissue complications and impaired knee function^1–3^. Because of the high-energy shearing and compressive stress exerted on the knee joint, menisci and ligaments are at considerable risk for injury, the overall incidence of which has been reported to be 39% to 99% and 16.7% to 57%, respectively^1,3–10^. Several authors have reported that soft tissue injury occurred more frequently with increasing displacement of tibial plateau fractures^11–14^. However, previous retrospective studies relied on data mostly obtained from MR images or arthroscopic findings. To our knowledge, there has been no prospective study to investigate the associated meniscal and cruciate ligament injuries associated with tibial plateau fractures as documented by diagnostic knee arthroscopy in a large sample of patients.

At the end of 2015, we began to perform percutaneous treatment for tibial plateau fractures using the self-designed traction device. Because a traditional arthrotomy was not performed with this technique, arthroscopic examination was performed for each patient in order to evaluate for intra-articular soft tissue injures. The purpose of this prospective study was twofold: (1) to determine the incidence of meniscal and cruciate ligament injuries in tibial plateau fractures detected by diagnostic arthroscopy, and (2) to correlate these soft tissue injuries with measurements of displacement on computed tomography (CT) images, in order to indentify radiological predictors of associated meniscal and cruciate ligament injury.

## Materials and Methods

This is a prospective study done from January 2016 to February 2017 at our academic level 1 trauma center. All skeletally mature patients who had a closed tibial plateau fracture with indications for operative treatment were enrolled into this prospective protocol. There was no maximum age restriction. Patients with substantial metabolic bone disease, pathological fractures, isolated intercondylar eminence fractures, extra-articular proximal tibial fracture, or any life-threatening condition were excluded. In order to eliminate the effects caused by other injuries, patients with another ipsilateral peri-articular fracture (i.e., distal femoral fracture, femoral condyle fracture or patellar fracture) or a previous proximal tibial fracture were also excluded. The indications for operative fixation included tibial plateau fractures with a varus or valgus malalignment of 10 degrees or greater between the injured and uninjured sides, and an intra-articular step-off at least 3 mm or a condylar widening greater than 5 mm measured on scaled radiographs and CT scans.

Full approval from the ethics committee of the Third hospital of Hebei Medical University (NO. KE 2016-001-1) was received for this study and all subjects enrolled provided informed consent. A total of 100 consecutive patients were included in this study. There were 77 men and 23 women, with 25 left knee injuries and 77 right knee injuries. Two patients suffered from bilateral tibial plateau fractures. The average age in this series was 44.60 ± 13.28 years (range 18–72). A total of 48 patients were injured due to a fall, 42 patients were injured in a traffic accident, 7 patients sustained crush injuries, and 3 patients sustained sprain injuries. 26 patients had other associated skeletal injuries, none of which occurred around the injured knee.

### Radiological measurements

**Figure 1:**
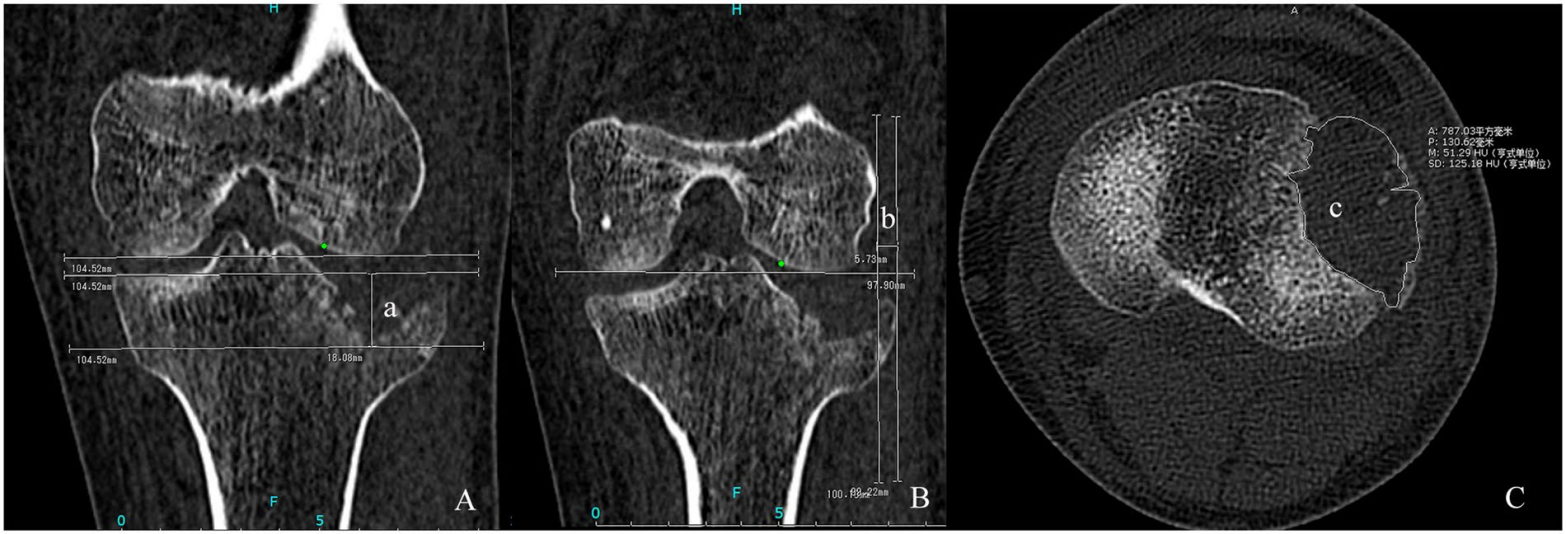
A 42-year-old male who sustained a type II fracture of tibial plateau. (**A**) Coronal reformatted CT image, a = the depth of lateral plateau depression, 18.08 mm; (**B**) Coronal reformatted CT image, b = lateral plateau widening, 5.73 mm; (**C**) Axial view of CT image, c = the area of lateral depression, 787.03 mm^2^.

Preoperative radiographic examinations consisted of plain radiographs and multidetector computed tomography (MDCT) scans. MDCT was indicated for detailed evaluation of the fracture morphology which facilitated surgical plan development. MDCT This was performed on a 64 slice multi-detector CT scanner (Siemens Sensation 64, Erlangen, Germany) using the following scan parameters: detector collimation 64 × 0.6 mm, tube voltage 120 kV scanning in a caudo-cranial direction. All radiological information was independently assessed by two authors (Zheng. and Chang.), who assigned the Schatzker classification and made the CT measurements using the PACS (Picture Archiving and Communication Systems) Imaging System. The mean values of fracture displacement measured by the two authors were used in the analysis. For articular depression and widening displacement, measurements were assessed from the coronal reformatted plane which was tangent to the posterior edge of femoral condyles in the transverse view, and parallel to the longitudinal axis of the tibial shaft in the sagittal view. The maximum amount of articular depression was measured from the intact plateau line (parallel to the femoral condyles) to the lowest point of depressed subchondral bone (Fig. 1A). Using the femoral condyle as a reference, widening displacement was equal to the distance between the tangental line to the femoral epicondyle (perpendicular to the femoral condyles) and the most laterally displaced point of tibial plateau (Fig. 1B). The area of depressed region was calculated from the CT scan coronal reformatted plane which could manifest the anterior and posterior edge of the depression region, the appropriate section could be easily identified to calculate the depression area using the freehand ROI (region of interest) tool (Fig. 1C). All parameters were calculated in millimeters and independently measured for the medial and lateral plateau.

### Surgical technique

**Figure 2:**
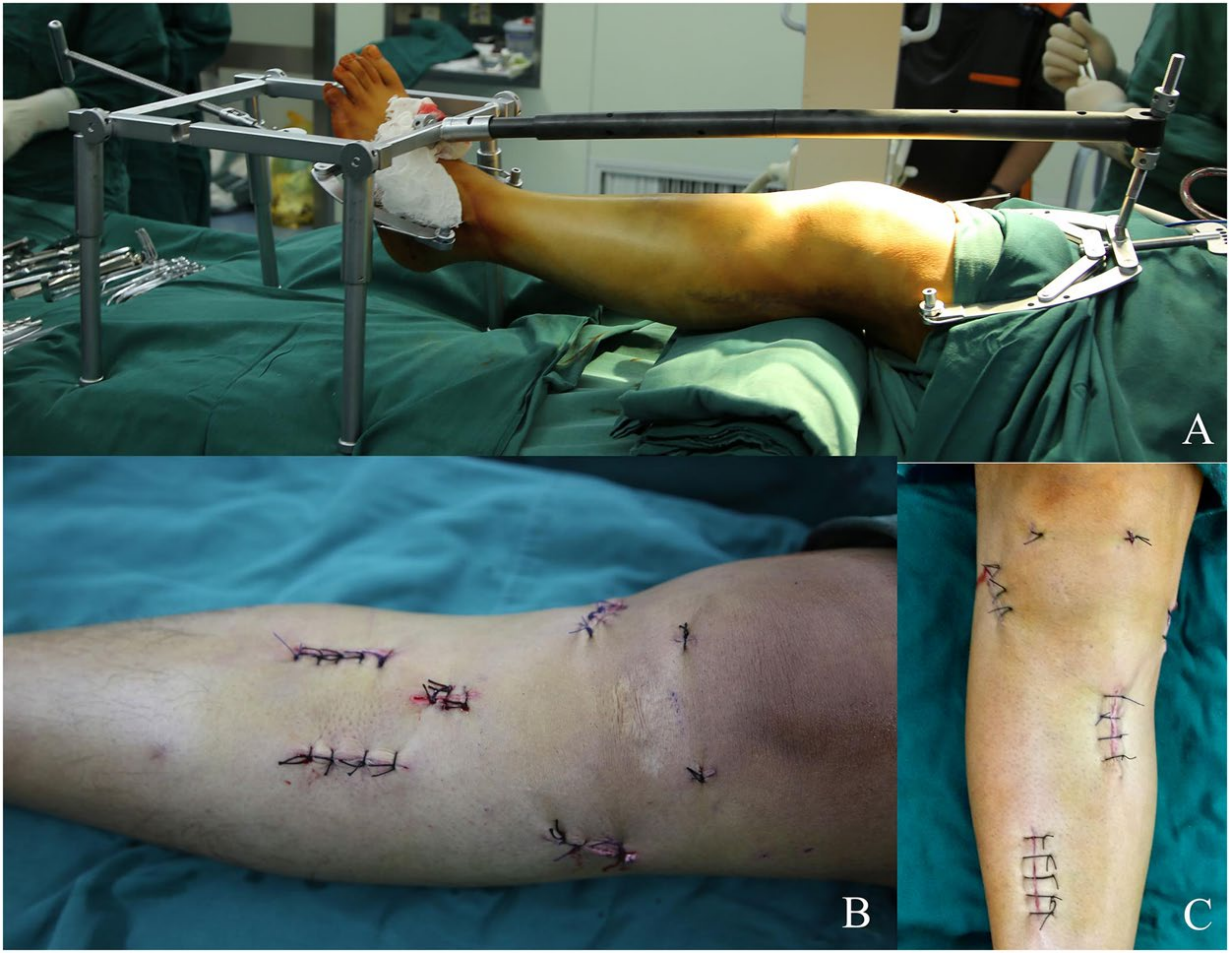
Closed reduction and internal fixation using bidirectional rapid reductor. (**A**) Intra-operative view of bidirectional rapid reductor; (**B**) The incisions of the patient who sustained a type VI fracture; (**C**) The incisions of the patient who sustained a type II fracture.

All fractures underwent closed reduction and internal fixation with the use of the bidirectional rapid reductor^15^ (Fig. 2). Patients were placed in the supine position on a radiolucent operating table with a tourniquet placed around the proximal thigh. The tourniquet was inflated to 300 mmHg once the closed traction was complete. A rolled sheet was placed under the affected knee joint to maintain knee flexion at 30 degrees. After confirming that all parts of the reductor were securely joined, a closed-loop traction system was formed by connecting the distal femur, knee joint and distal tibia to the reductor. Through ligamentotaxis and longitudinal traction, the tibial plateau’s widening, tibial length as well as varus or valgus angulation could be primarily corrected under fluoroscopic guidance. After achieving satisfactory reduction, confirmed by C-arm examination both on anteroposterior (AP) and lateral views, the reductor could sustain the lower extremity in a reduced position. Care was taken to locate the orientation of depressed fragments and a 2.5 mm Kirschner was first drilled under the fluoroscopic view to the center of depressed fragments. The ideal entry point was 2 cm below the centre of tibia tubercle, slightly medial or lateral to the long axis of tibia which depends on the location of depressed fragments. Then, a cortical portal was made, and the depressed fragments was elevated with a bone tamp via the inferior transosseous tunnel created by step drills. The whole reduction process is performed with gentle tapping of the bone tamp under the inspection of AP and lateral views of fluoroscopy. Autogenous bone graft harvested from the iliac crest was then inserted into the bone tunnel to support the subchondral bone and articular surface.

After the accomplishment of internal fixation, a systematic arthroscopic examination was performed. Standard anterolateral and anteromedial portals were created for insertion of the arthroscope as well as working instruments. Inflow irrigation was created with the use of gravity. After the evacuation of the hemarthrosis and other debris, all intra-articular structures were thoroughly evaluated and probed as necessary so that no injuries are overlooked. The sequence of structures to be examined were suprapatellar pouch, patellofemoral joint, femoral notch (ACL and PCL), medial compartment (medial meniscus and articular surface) and lateral compartment (lateral meniscus and articular surface). The concept of meniscus contusion was firstly adopted in 2001 by Cothran *et al*.^16^. We described this pathology as a traumatic-related injury that usually showed evidence of congestive appearance of the peripheral menisci in the absence of frank tearing or a significant loss of stability. Arthroscopic evaluations were all performed by the senior author (Shao.) with expertise in sports medicine.

Conservative treatment was performed for patients with meniscal contusion. Patients with complete meniscal tear were treated with suture repair or partial meniscectomy. Considering the background of a severe bony injury, for patients with cruciate ligament injury, we followed the “active neglect” policy and performed a conservative treatment initially with planned repair in a secondary phase if necessary^17^. Arthroscopic findings for associated soft tissue injuries were recorded and then correlated to the measurements obtained from CT scans with regard to Schatzker classification and bony displacement of tibial plateau.

### Statistical Methods

Statistical analyses were all performed using IBM SPSS (Version 19.0, SPSS, Inc., and Chicago, IL). The percentage of each soft-tissue injury relative to the total number of fractures was first calculated, and then compared with the Schatzker classification to analyze any significant predilection using Fisher exact test. Patients were divided into 2 groups based on whether they sustained a meniscus or cruciate ligament lesion. The independent variables include age, sex, energy level of injury (Schatzker type I to III were classified as the low-energy fracture patterns and Schatzker type IV to VI were classified as the high-energy fracture patterns)^13,18,19^, the depth of lateral plateau depression (dLPD), the area of lateral plateau depression (aLPD), lateral plateau widening (LPW), the depth of medial plateau depression (dMPD), the area of medial plateau depression (aMPD), and medial plateau widening (MPW). Univariate analyses were conducted to identify association of each soft tissue injury with potential predictors using Fisher exact test and t tests. Mann-Whitney U test was used for continuous variables with abnormal distribution. Receiver operating characteristic (ROC) analysis was performed to determine the ability and cutoff point for each potential variable to predict the presence of soft-tissue injuries. Logistic regression was performed to assess the impact of all potential predictors (significant in the univariate analysis) on soft-tissue injuries in multivariate model. Descriptive statistics were reported as percentage or mean ± SD (SD, standard deviation). For all tests, significance was considered as *P* < 0.05.

### Ethical Board Review statement

The Ethical Board Review of the Third Hospital of Hebei Medical University (Shijiazhuang, China) has approved the conduction of the study after a thorough examination and verification. The study has been performed in accordance with the ethical standards of Declaration of Helsinki in the 1964.

The study was registered in Chinese Clinical Trial Registry and the registration number: ChiCTR-OPC-16008012.

## Results

According to Schatzker classification^2^, distribution of fracture types was as follows: 4 type I (pure split), 33 type II (split combined with depression), 12 type III (pure depression), 22 type IV (medial plateau), 16 type V (bicondylar) and 15 type VI (plateau and metaphysis) fractures. Of the 102 fractures, 70 (68.2%) showed evidence of intra-articular lesions through arthroscopic evaluation. According to the tear patterns and configurations, meniscal pathology could be described as simply contusion, peripheral tear, radial tear, flap tear, bucket-handle tear, horizontal tear and degenerative changes^20^. The first six injuries were attributed to traumatic group, the overall incidence of which was 52.9% (54/102) with 90.7% (49/54) of these being lateral, and 16.6% (9/54) medial, while 7.4% had bilateral meniscal tears. Peripheral tear was the most common type of meniscal injury which was noted in 24 fractures and was most frequent in lateral meniscus. The specific incidence of other meniscal injuries are reported in Table 1. Based on Schatzker classification, type VI fractures had significantly higher rate of bucket-handle tear than other fracture types (*P* = 0.04) (Table 2). Injuries to the anterior cruciate ligament (ACL) or posterior cruciate ligament (PCL) were classified as an avulsion fracture, partial tear, and complete tear; this occurred in 23 of the 102 fractures (22.5%). Specifically, 15 (14.7%) fractures had evidence of ACL pathology, and 10 (9.8%) fractures had evidence of PCL pathology. Avulsion fracture was the most common type of cruciate ligament injury which occurred in 9.8% of all fractures (Table 1). No significant difference existed between fracture type and incidence of ACL or PCL injuries (Table 3).

**Table 1:** Arthroscopic findings on abnormal menisci and cruciate ligament.

Soft tissue injury (n = 70)		Side	Number (n)	Incidence (%)
Menisci	Contusion	Lateral	13	12.70%
(n = 62, 60.8%)	(n = 15, 14.7%)	Medial	2	2.00%
	Peripheral Tear	Lateral	21	20.60%
	(n = 24, 23.5%)	Medial	3	3.00%
	Radial Tear	Lateral	5	5.00%
	(n = 7, 7.0%)	Medial	2	2.00%
	Flap Tear	Lateral	4	4.00%
	(n = 4, 4.0%)	Medial	0	0
	Bucket-handle tear	Lateral	9	9.00%
	(n = 9, 9.0%)	Medial	0	0
	Horizontal tear	Lateral	0	0
	(n = 2, 2.0%)	Medial	2	2.00%
	Degenerative changes	Lateral	4	4.00%
	(n = 9, 9.0%)	Medial	8	8.00%
Cruciate ligament	Avulsion Fracture	ACL	10	9.80%
(n = 23, 22.5%)	(n = 10, 10.0%)	PCL	1	1.00%
	Partial Tear	ACL	3	3.00%
	(n = 7, 7.0%)	PCL	4	4.00%
	Complete Tear	ACL	2	2.00%
	(n = 7, 7.0%)	PCL	5	5.00%

ACL anterior cruciate ligament, PCL posterior cruciate ligament.

**Table 2:** Incidence of traumatic meniscus injuries based on fracture pattern.

Meniscal pathology	Schatzker classification						*p*
	I(n = 4)	II(n = 33)	III(n = 12)	IV(n = 20)	V(n = 18)	VI(n = 15)	
Contusion	0	6	1	4	2	2	0.95
Peripheral Tear	1	9	3	5	4	2	0.95
Radial Tear	0	3	0	2	1	1	0.95
Flap Tear	0	2	0	0	1	1	0.88
Bucket-handle tear	0	0	0	1	3	5	0.04*
Horizontal tear	0	1	0	0	1	0	0.87
Total injuries	1	21	4	12	12	11	0.22

Significance is denoted by * at the 0.05 level.

**Table 3:** Incidence of cruciate ligament injuries based on fracture pattern.

Ligament pathology	Schatzker classification						*p*
	I(n = 4)	II(n = 33)	III(n = 12)	IV(n = 20)	V(n = 18)	VI(n = 15)	
ACL Avulsion Fracture	2	1	1	3	1	2	0.08
ACL Patial Tear	0	1	0	1	0	1	0.87
ACL Complete Tear	0	0	0	0	2	0	0.14
PCL Avulsion Fracture	0	0	0	1	0	0	0.68
PCL Patial Tear	0	3	0	0	0	2	0.35
PCL Complete Tear	0	2	0	2	1	0	0.87
Total injuries	2	7	1	7	4	5	0.39

**Figure 3:**
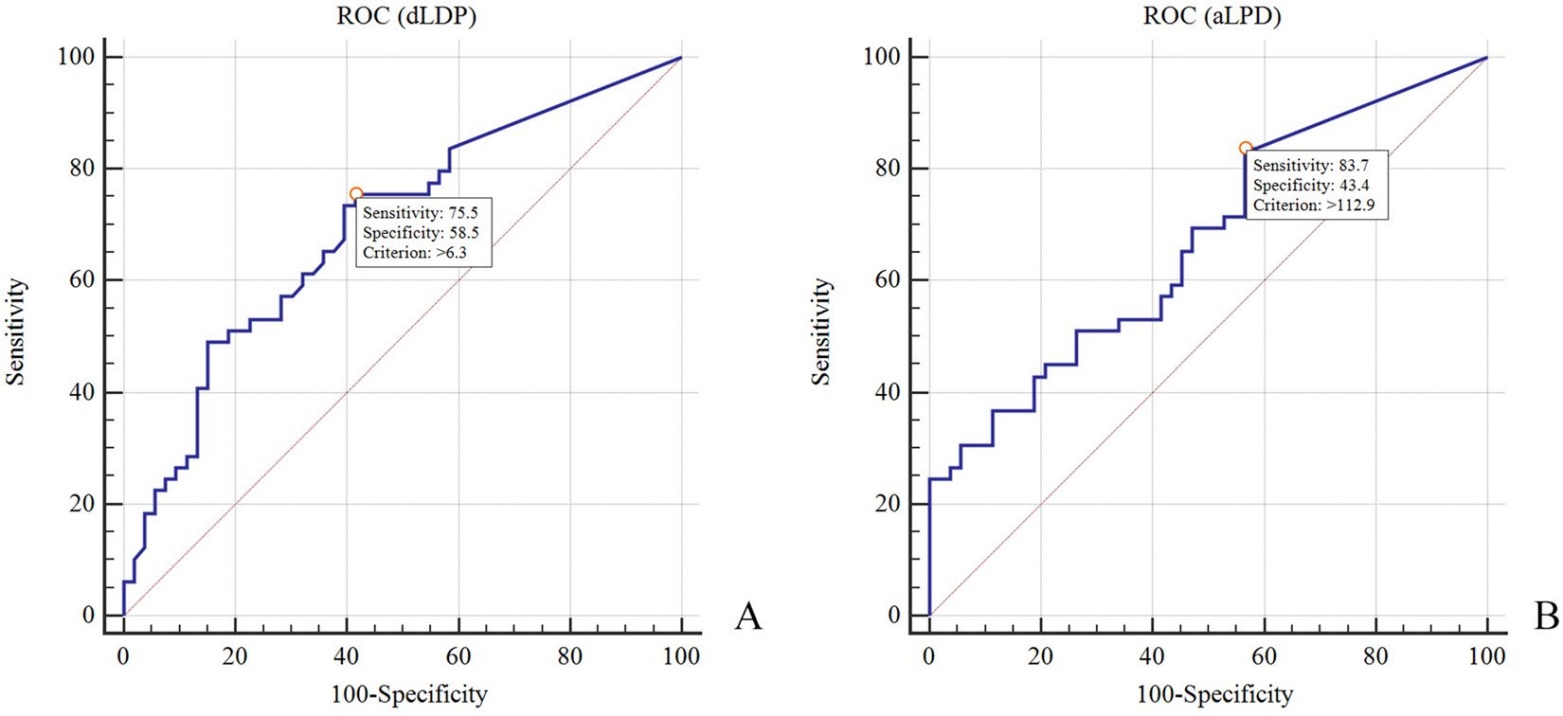
(**A**,**B**) dLDP and aLPD as models for predicting lateral meniscal injuries.

Univariate analyses showed no significant differences in age, sex, or energy level of injury in patients with and without meniscal and cruciate ligament pathology (Tables 4–7). The depth and area of lateral plateau depression (dLPD and aLPD) were significantly greater among patients with a lateral meniscal lesion as compared to those without an injury (Table 4). The ROC analyses indicated that the most appropriate cutoff points for dLPD and aLPD were 6.3 mm (sensitivity 75.5%, specificity 58.5%, AUC 0.70) and 112.9 mm^2^ (sensitivity 83.7%, specificity 43.4%, AUC 0.67) respectively (Fig. 3). In the logistic regression model, only dLDP showed to be the significant predictor of a lateral meniscus tear (Table 8).

**Table 4:** Differences in patient demographic and radiological characteristics by lateral meniscus lesion.

Characteristics	lateral meniscus lesion		*p*
	Yes (n = 49)	No (n = 53)	
Mean age, y	43.5 ± 12.2	43.2 ± 14.0	0.71
**Sex, %**			
Male	37 (75.5)	42 (79.2)	0.65
Female	12 (24.5)	11 (20.8)	
**Energy level of injury, %**			
Low	23 (46.9)	26 (49.1)	0.83
High	26 (53.1)	27 (50.9)	
dLPD, mm	11.0 ± 7.9	6.0 ± 6.3	<0.01*
aLPD, mm^2^	433.7 ± 299.1	258.6 ± 250.9	<0.01*
LPW, mm	7.0 ± 7.4	4.6 ± 4.3	0.07
dMPD, mm	0.3 ± 1.5	0.4 ± 1.5	0.49
aMPD, mm^2^	9.9 ± 61.0	20.5 ± 83.7	0.45
MPW, mm	0.1 ± 0.8	0.2 ± 1.0	0.35

dLPD depth of lateral plateau depression, aLPD area of lateral plateau depression, LPW lateral plateau widening, dMPD depth of medial plateau depression, aMPD area of medial plateau depression, MPW medial plateau widening. *p < 0.05.

**Table 5:** Differences in patient demographic and radiological characteristics by medial meniscus lesion.

Characteristics	medial meniscus lesion		*p*
	Yes (n = 9)	No (n = 93)	
Mean age, y	47.6 ± 14.1	42.9 ± 13.0	0.31
**Sex, %**			
Male	6 (66.7)	73 (78.5)	0.65
Female	3 (33.3)	20 (21.5)	
**Energy level of injury, %**			
Low	2 (22.2)	47 (50.5)	0.16
High	7 (77.8)	46 (49.5)	
dLPD, mm	7.5 ± 10.5	8.5 ± 7.2	0.35
aLPD, mm^2^	221.2 ± 286.0	354.5 ± 286.4	0.18
LPW, mm	7.8 ± 8.4	5.5 ± 5.1	0.62
dMPD, mm	2.2 ± 3.4	0.2 ± 1.0	<0.01*
aMPD, mm^2^	120.0 ± 198.2	5.3 ± 36.7	<0.01*
MPW, mm	0.9 ± 2.0	0.1 ± 0.8	0.03*

*p < 0.05.

**Table 6:** Differences in patient demographic and radiological characteristics by ACL lesion.

Characteristics	ACL lesion		*p*
	Yes (n = 15)	No (n = 87)	
Mean age, y	42.5 ± 13.5	43.5 ± 13.1	0.8
**Sex, %**			
Male	14 (93.3)	65 (74.7)	0.2
Female	1 (6.7)	22 (25.3)	
**Energy level of injury, %**			
Low	5 (33.3)	44 (50.6)	0.22
High	10 (66.7)	43 (49.4)	
dLPD, mm	5.9 ± 6.8	8.8 ± 7.6	0.17
aLPD, mm^2^	203.0 ± 242.2	366.8 ± 289.0	0.045*
LPW, mm	5.9 ± 6.8	5.1 ± 4.7	0.026*
dMPD, mm	0.7 ± 1.8	0.3 ± 1.4	0.21
aMPD, mm^2^	15.7 ± 46.1	15.4 ± 77.5	0.21
MPW, mm	0.4 ± 1.5	0.1 ± 0.7	0.53

*p < 0.05.

**Table 7:** Differences in patient demographic and radiological characteristics by PCL lesion.

Characteristics	PCL lesion		*p*
	Yes (n = 10)	No (n = 92)	
Mean age, y	42.0 ± 15.5	43.5 ± 12.9	0.74
**Sex, %**			
Male	6 (60.0)	73 (79.3)	0.22
Female	4 (40.0)	19 (20.7)	
**Energy level of injury, %**			
Low	5 (50.0)	44 (47.8)	1.0
High	5 (50.0)	48 (52.2)	
dLPD, mm	8.0 ± 9.8	8.4 ± 7.3	0.56
aLPD, mm^2^	252.0 ± 79.7	291.1 ± 30.3	0.36
LPW, mm	4.7 ± 4.1	5.9 ± 5.6	0.76
dMPD, mm	0.4 ± 1.4	0.3 ± 1.5	0.61
aMPD, mm^2^	17.1 ± 54.2	15.2 ± 75.6	0.57
MPW, mm	1.1 ± 2.4	0.1 ± 0.4	<0.01*

*p < 0.05.

**Table 8:** Predictors of lateral meniscus lesion from multivariate logistic regression.

Characteristics	OR	95%CI	*p*
dLPD	4.35	1.86–10.17	0.001*
aLPD	—	—	0.622

CI, confidence interval for odds ratio (OR), —, data not available. *p < 0.05.

As for medial meniscal lesion, dLPD, aLPD and MPW were significantly greater among patients with a medial meniscal lesion (Table 5). However, of all 102 fractures, only six fractures were detected with a medial plateau depression and four patients had medial plateau widening. ROC analysis demonstrated no clear threshold for these three radiological parameters which yielded a combination of high sensitivity and specificity. Therefore, each fracture was assigned to one of two categories according to whether the plateau had a medial displacement or plateau depression. The categorical independent variables were entered into the multivariable logistic regression analysis. Logistic regression analysis indicated that tibial plateau fractures with medial depression were likely to develop medial meniscal lesion after adjusting for medial plateau widening (odds ratio 9.53; 95% CI 1.31–69.2).

**Figure 4:**
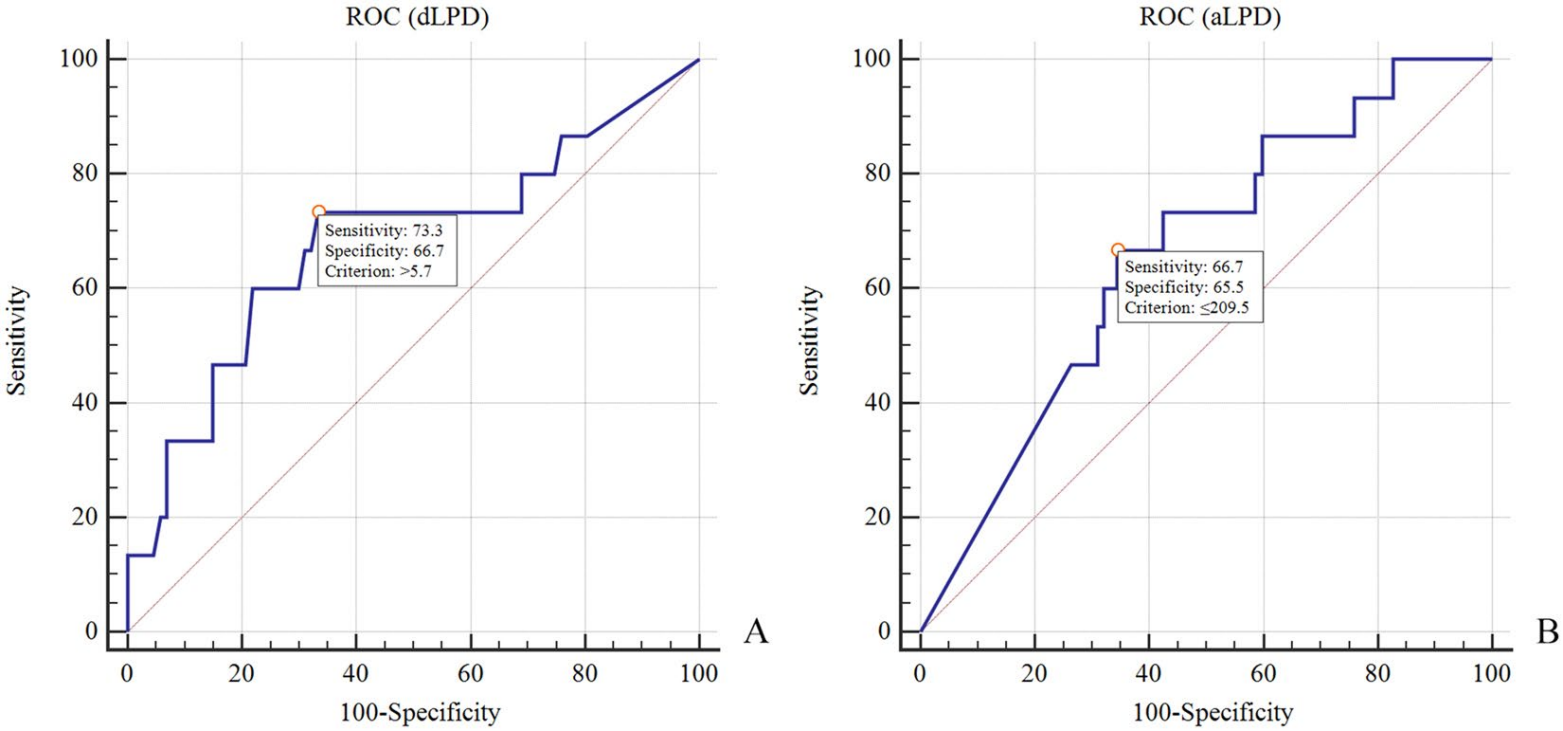
(**A**,**B**) aLPD and LPW as models for prediction ACL injuries.

Univariate analysis showed lesser aLPD and greater LPW were found among patients with an ACL lesion compared with patients without a lesion (Table 6). The receiver operating characteristic analyses indicated that the most appropriate cutoff points for aLPD and LPW was 209.5 mm^2^ (sensitivity 66.7%, specificity 65.5%, AUC 0.66) and 5.7 mm (sensitivity 75.5%, specificity 58.5%, AUC 0.68) for detecting an ACL lesion (Fig. 4). In the logistic regression model, both aLPD and LPW were significant predictors of an ACL lesion (Table 9). As for PCL, univariate analysis showed that the amount of MPW were significantly greater among patients with a PCL lesion compared with patients without a lesion (1.1 ± 2.4 mm^2^ vs. 0.1 ± 0.4 mm^2^; *P* = 0.005) (Table 7).

**Table 9:** Predictors of ACL lesion from multivariate logistic regression.

Characteristics	OR	95%CI	*p*
aLPD	6.81	1.80–25.76	0.005*
LPW	9.22	2.33–36.54	0.002*

CI, confidence interval for odds ratio (OR). *p < 0.05.

## Discussion

The results of our study demonstrate a high prevalence of meniscal injuries (52.9% of subjects) associated with operative tibial plateau fractures. Similar results have been reported in at least six other clinical studies. The prevalence of associated meniscal injuries ranged from 38.9% to 99.0%, depending on the study^1,7,10,18,21,22^. We found the incidence of degenerative pathology of the meniscus in 9 (9.0%) of 102 fractures and mostly happened on medial meniscus. None of previous studies differentiated such degenerative changes from actual traumatic-related lesion. This might be a potential reason why incidence of meniscus injury was much higher in some studies that based their diagnosis on MRI findings alone^1,7^.

Our results also revealed that a significantly higher incidence of bucket-handle tears, which was more commonly observed in Schatzker VI fractures compared with other fracture types. This kind of injury represents an unstable tear and all occurred in the lateral meniscus and in high energy fractures (Schatzker IV to VI). We noticed that even when fractures were completely reduced, inner fragment of the meniscus tear could hardly be reduced or repaired. Therefore, we performed total or partial resection in most circumstance. Our suggestion is that patients who sustained a high-energy fracture especially when metaphysis was involved were more likely to have bucket-handle meniscal tears, and arthroscopic examination is highly recommended for accurate diagnosis.

In our study, ACL lesions were diagnosed at arthroscopy in 14.7% of fractures. The incidence was lower than the proportion of ACL lesions reported in previous studies using ARIF or MRI. Gardner *et al*. firstly documented the incidence of soft-tissue injuries on MRI in 103 patients. They noted that 57% of their patients had destabilizing ACL injuries (footprint avulsion or complete tear)^7^. Tang *et al*. retrospectively analyzed the soft-tissue injuries in 132 patients undergoing ARIF. They reported a 6.8% rate of ACL tears, and a 37.1% rate of ACL avulsion fractures^18^. Compared to previous findings, the incidence of complete cruciate ligament tear was relatively low (7.0%) in our series, and this kind of injury often occurred in the substance of the PCL. According to the three-zone concept^23^, PCL tear mostly occurred in zone 1 which extends from the PCL femoral insertion to where that ligament disappears. No significant correlation was found in terms of cruciate ligament injuries and different types of fracture. In contrast, Stannard *et al*. found that in their series of 103 patients cruciate ligament injuries happened more frequently in high-energy tibial plateau fractures (Schatzker type IV to VI)^19^. Abdel-Hamid *et al*. also found that the incidence of ACL injury was significantly higher in Schatzker IV and VI fractures compared with other types^21^. These different findings may be attributed to the relatively low number of cruciate ligament injuries identified in the present study.

Several studies have reported correlations between radiological assessments and soft-tissue injuries in lateral tibial plateau fractures^11–14,24^. Gardner *et al*. reported that lateral meniscal injury occurred in 83% of fractures when depression was greater than 6 mm and widening was greater than 5 mm^11^. Ringus *et al*. noted that the degree of articular depression was a predictor of meniscus pathology in tibial plateau fractures^12^. They measured the depth of lateral plateau depression on coronal CT images of patients undergoing open reduction and internal fixation. An eight-fold increase in risk of lateral meniscus tear was observed if the depression was >10 mm. However, the studies mentioned above all used MRI instead of diagnostic arthroscopic findings to detect intra-articular injuries and mostly focused solely on lateral plateau and meniscal injuries. Tang *et al*. first demonstrate the correlation of preoperative imaging parameters with arthroscopic findings in acute tibial plateau fractures. Similarly, they found that lateral tibial plateau depressions >11 mm were significantly associated with increased risk of lateral meniscus tears^18^. However, this study was retrospective, collecting the data from 2005 to 2015. The authors also emphasized the correlation between lateral plateau displacement and lateral meniscus tears.

In contrast to the prior studies, this study was prospectively designed and both medial and lateral plateau were included for analysis. Furthermore, we introduced the parameter of total depression area as a variable to predict associated soft tissue injuries together with depression depth and plateau widening. This study revealed that patients with >6.3 mm depression had a four-fold increased risk of sustaining a lateral meniscus lesion; aLPD, however, was not statistically significant for lateral meniscus lesion after multivariable regression. We can thus hypothesize that there might be an issue of collinearity between aLPD and dLPD. When using the stepwise regression model, dLPD became the most suitable variable which could predict the lateral meniscus injury. We did not identify a threshold for bony displacement of the medial plateau (aMPD, dMPD and MPW) which can significantly predict the occurrence of medial meniscus pathology. Therefore, we divided the fractures into two categories according to whether the plateau had a medial displacement or plateau depression. The results of logistic regression revealed that patients with medial plateau depression had an almost ten-fold increased risk of sustaining a medial meniscus tear than patients without medial depression. Our results showed that both lateral and medial meniscal injuries were closely related to tibial plateau depression, which indicated that articular depression was a potential predictor of meniscal injuries in operative tibial plateau fractures.

In addition, we observed an increased incidence of ACL injuries associated with a greater degree of LPW and smaller aLPD. Our findings were remarkably different from previous studies. Previous studies have shown that valgus moments on the knee joint have been suggested as a risk factor for anterior cruciate ligament (ACL) injuries during single-leg landing^25–27^. We hypothesized that lateral plateau widening might represent the energy of valgus stress applied on knee joint. Therefore, the greater the energy applied on the lateral tibial plateau, the greater the degree of lateral widening and the greater the incidence of ACL lesion can be expected. The area of tibial plateau depression, on the other hand, represented the energy of axial loading transmitted from femoral condyles to the tibial plateau. Hence patients with smaller aLPD might sustain much shear force or valgus stress rather than compression force, which could easily result in ACL pathology if the ACL substance or ACL insertion is compromised. Another potential explanation is that fractures with obvious plateau widening and moderate depression area were often classified as Schatzker type V or VI. Patients that sustained a Schatzker type V or VI fractures would be prone to sustain an ACL lesion. Lacking evidence of biomechanical trials, these findings should be further explored in cadaveric models.

Despite of a prospective design, this study still has some limitations. First, patients with minimally displaced tibial plateau fractures were not included in our protocol. Second, all arthroscopic examinations were performed after the accomplishment of closed reduction and internal fixation, which may not accurately reflect the soft tissues injuries shortly after fractures. The anatomic situation could have been altered when depressed fragments were reduced using a bone tamp, especially for meniscus tears. Finally, limited surgical vision from the anterior portal was not enough for thorough examination for PCL lesion. The substantive portion and tibial insertion of PCL could be only examined using a probe, and this may affect accuracy of the diagnosis.

## Conclusion

Associated meniscal and ligament pathologies were commonly seen among patients who sustained tibial plateau fractures. Schatzker type VI fractures had a significantly higher rate of bucket-handle tears than other fracture types. No significant difference existed between fracture type and incidence of ACL or PCL injuries. This study also revealed that increased depth of lateral depression (>6.3 mm) was associated with higher rate of lateral meniscus lesion; a higher rate of ACL lesion was observed when the area of lateral depression was ≤209.5 mm^2^ and when lateral widening was >5.7 mm. Therefore, preoperative CT measurements might help predict a higher risk of meniscus and ACL pathology, and guide the surgeon to carefully examine these structures and be prepared to treat these associated injuries.
